# Networked Cages for Enhanced CO_2_ Capture and Sensing

**DOI:** 10.1002/advs.201800141

**Published:** 2018-05-17

**Authors:** Zhen Wang, Hui Ma, Tian‐Long Zhai, Guang Cheng, Qian Xu, Jun‐Min Liu, Jiakuan Yang, Qing‐Mei Zhang, Qing‐Pu Zhang, Yan‐Song Zheng, Bien Tan, Chun Zhang

**Affiliations:** ^1^ College of Life Science and Technology National Engineering Research Center for Nanomedicine Huazhong University of Science and Technology Wuhan Hubei 430074 China; ^2^ School of Chemistry and Chemical Engineering Huazhong University of Science and Technology Wuhan Hubei 430074 China; ^3^ School of Materials Science and Engineering Sun Yat‐Sen University Guangzhou 510275 China; ^4^ School of Environmental Science and Technology Huazhong University of Science and Technology Wuhan Hubei 430074 China

**Keywords:** aggregation‐induced emission, cage compounds, carbon dioxide capture, carbon dioxide sensors, porous polymers

## Abstract

It remains a great challenge to design and synthesize a porous material for CO_2_ capture and sensing simultaneously. Herein, strategy of “cage to frameworks” is demonstrated to synthesize fluorescent porous organic polymer (pTOC) by using tetraphenylethylene‐based oxacalixarene cage (TOC) as the monomer. The networked cages (pTOC) have improved porous properties, including Brunauer–Emmett–Teller surface area and CO_2_ capture compared with its monomer TOC, because the polymerization overcomes the window‐to‐arene packing modes of cages and turns on their pores. Moreover, pTOC displays prominent reversible fluorescence enhancement in the presence of CO_2_ in different dispersion systems and fluorescence recovery for CO_2_ release in the presence of NH_3_·H_2_O, and is thus very effective to detect and quantify the fractions of CO_2_ in a gaseous mixtures.

Organic molecular cages (OMCs)^1^, ^2^, ^3^ have attracted great attention in the field of porous organic materials because of their intrinsic porosity and the solution‐processible properties. For example, Cooper and co‐workers applied imine cages^4^ for gas storage and separation for the first time. Mastalerz and co‐workers^5^ synthesized a triptycene‐based cage with high specific surface area of 3758 m^2^ g^−1^. Zhang's OMCs^6^ exhibited exceptional high absorption selectivity (138/1) for CO_2_ over N_2_. However, the pores of OMCs are often blocked by their window‐to‐arene assembled patterns.[[qv: 4a,c]] Moreover, many cages' porosity is generally lost with the removal of solvent molecules in the process of heat treatment.[[qv: 4d,5c]] To retain the permanent porosity of OMCs, coupling these OMCs building blocks covalently into the polymeric frameworks to turn on their pores may be a promising strategy. However, such cage‐based polymeric frameworks are rarely reported. Cooper's[[qv: 7a]] and Huang's group[[qv: 7b]] reported two porous‐networked cages by coordinative bonds with zinc or sodium ions, but their instability limited their applications in acidic or basic conditions. Although Zhang's cage‐based frameworks[[qv: 6b]] linked by covalent bonds overcame the shortcoming of stability problem to some extent, its surface area is too low to be used for practical applications.

Growing concerns over carbon dioxide (CO_2_) emission have been raised not only because of its role to enhance greenhouse effect,^8^ but also its harmful effect on chemical industries, human health, and the protection of historic relics, which has prompted researchers to develop effective materials that can be used for CO_2_ capture and sensing simultaneously.^9^ Despite a significant progress in the development of various CO_2_ sorbents based on porous materials, it is still a formidable challenge to develop new porous materials that can detect and capture CO_2_ simultaneously. Recently, Yuan^10^ reported nitrogen‐doped porous carbon with high CO_2_ uptake and resistive sensitivity for the detection of CO_2_, but the pursuit of new materials that can be used as more efficient CO_2_ sorbents and detect of CO_2_ with fluorescent technology^11^ remains a critical issue.

Recently, we reported the synthesis of a porous tricyclooxacalixarene emissive cage by nucleophilic aromatic substitution reaction (S_N_Ar).[[qv: 12a]] X‐ray crystallographic analysis and nitrogen sorption experiments both confirmed the presence of micropores with an average size of 5.8 Å, which is comparable to the size of CO_2_ (3.3 Å). From these results, we predicted that if CO_2_ molecules are trapped in the intrinsic cavity of OMCs, the rotation and vibration of phenyl rings in tetraphenylethylene (TPE) units may be affected resulting in fluorescence enhancement because of the aggregation‐induced emission (AIE) effect,^13^, ^14^, ^15^, ^16^ and may offer an alternative approach to sense CO_2_. Herein, we synthesized a cage‐based polymeric framework (pTOC) by nickel (0)‐catalyzed Yamamoto‐type Ullmann crosscoupling reaction (**Scheme** **1**)^17^ from TPE‐based oxacalixarene cage (TOC) having polymerizable groups. The strategy of networking cages by covalent coupling not only overcame the window‐to‐arene packing modes of cages to turn on their pores and displayed significantly enhanced Brunauer–Emmett–Teller (BET) surface area and CO_2_ capture capacity compared with that of TOC monomers, but also maintained cages' intrinsic fluorescent properties for CO_2_ sensing.

**Scheme 1:**
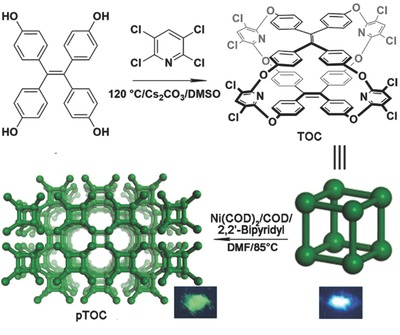
Graphical representation of synthesis of TOC and pTOC. The inset photographs of TOC and pTOC are excited under ultraviolet lamp (365 nm).

The oxacalixarene cage TOC was synthesized through one‐pot S_N_Ar reaction, as shown in Scheme 1. The S_N_Ar reaction of tetrahydroxy‐tetraphenylethylene 1 with 2, 3, 5, 6‐tetrachloro‐pyridine 2 in the presence of Cs_2_CO_3_ in dimethyl sulfoxide (DMSO) at 120 °C for overnight resulted in the formation of TOC (yield, 15%). With TPE units in cage scaffold, TOC not only emitted strong blue fluorescence in toluene (with the fluorescence quantum yield (*Φ*_f_) of 26%) but also displayed emissive behavior on aggregation in methanol/toluene (95/5, V/V) with *Φ*_f_ of 36% (Figures S2 and S3, Supporting Information), which was similar to our previous molecular cage.[[qv: 12a]]

The X‐ray single crystals analysis confirmed TOC's structure.^18^ By diffusing acetonitrile into toluene solution, the suitable single crystals of TOC were obtained. As shown in **Figure** **1**A,B, the two propeller‐like TPE units were fixed strictly by four pyridine units, which formed a quadrangular prismatic structure with distance between the top and bottom TPE of about 5.3 Å (Figure S4A, Supporting Information). In the solid state, TOCs pack in window‐to‐arene mode by interactions of C—H⋅⋅⋅O (*d*_H⋅⋅⋅O_ = 2.634 Å, θ_C—H⋅⋅⋅O_ = 138.89°), C—H⋅⋅⋅π (*d*_H⋅⋅⋅π_ = 2.888 Å), C—H⋅⋅⋅Cl (*d*_H⋅⋅⋅Cl_ = 2.911 Å, θ_C—H⋅⋅⋅Cl_ = 170.82°), and C—Cl···π (*d*_Cl⋅⋅⋅π_ = 3.384 Å) between two neighboring cages (Figure 1C; Figure S4B, Supporting Information). It is therefore that TOCs display no intercage window connectivity and form nonconnective lattice voids, and furthermore packing into nonporous framework, as illustrated by the blue Connolly surface (probe radius = 1.82 Å) (Figure 1D; Figure S5, Supporting Information).[[qv: 4a]] The N_2_ sorption experiment of TOC after desolvation at 120 °C for 10 h under vacuum showed almost no N_2_ uptake with BET surface area only 8 m^2^ g^−1^ (Figure S7A, Supporting Information), and confirmed the crystallographic measurements and atomistic simulations.

**Figure 1:**
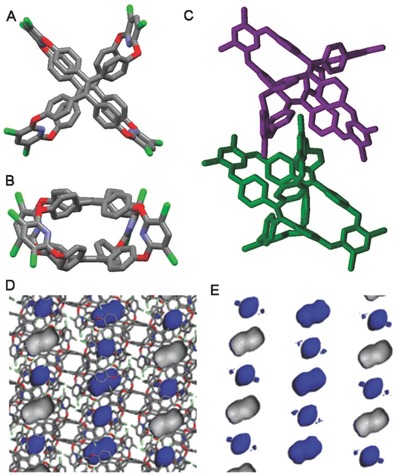
Top view A) and side view B) of X‐ray crystal structures of TOC. Two neighboring molecules of TOC pack in a window‐to‐arene mode (two neighboring TOC molecules were presented by different colors) C). The cross‐sectional images of the packing structures with D) and without E) cage framework show that TOC has nonconnective lattice voids, as illustrated by the blue Connolly surface (probe radius = 1.82 Å) applied to the crystal structure for the desolvated material.

To overcome the window‐to‐arene stacking and turn on the pores of TOC, we polymerized TOC into emissive cage‐based polymeric framework (pTOC) by Yamamoto‐type Ullmann crosscoupling reaction considering the polymerizable groups of chlorine directing toward outside the cage (Scheme 1 and Supporting Information). While comparing the Fourier transform infrared (FT‐IR) spectra of TOC and pTOC (Figure S6, Supporting Information), the aromatic C–Cl bending vibrations at 1091 cm^−1^ were disappeared when the monomers were transformed into polymers. Furthermore, the results of ^13^C crosspolarized magic angle spinning solid‐state (^13^C CP‐MAS) NMR confirmed the successful polymerization from TOC. For pTOC, the resonance peaks at δ = 156, 139, and 103 ppm could be assigned to aromatic carbons in the pyridine rings, and 119, 131, 139, and 151 ppm for the carbons in TPE moiety (**Figure** **2**A). The amount of Ni residue in pTOC was also measured as low as 0.08 wt% by inductively coupled plasma mass spectrometry (ICP‐MS). Transmission electron microscopy (TEM) and scanning electron microscopy (SEM) (Figure 2B,C) indicated the formation of rough particles of pTOC. The powder X‐ray diffraction experiment confirmed the noncrystalline nature of pTOC (Figure 2D). The thermogravimetric analysis (TGA) showed that pTOC was thermally stable without significant mass loss below 500 °C under nitrogen atmosphere (Figure 2E).

**Figure 2:**
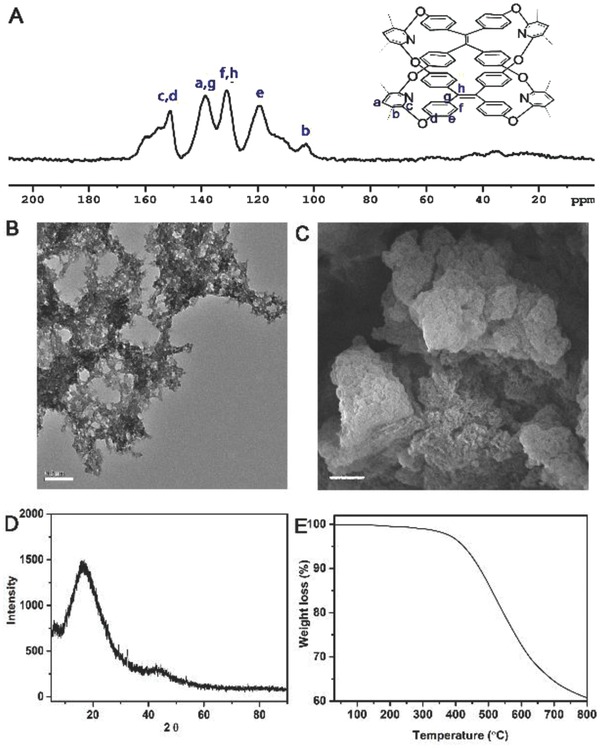
The ^13^C CP‐MAS NMR spectroscopy A), TEM image B) (bar = 0.2 µm), SEM image C) (bar = 2 µm), powder X‐ray diffraction D), and the TGA E) of pTOC.

To investigate the porous properties, the nitrogen sorption analysis of pTOC was conducted after desolvation at 120 °C for 10 h under vacuum. For pTOC, the BET surface area and pore volume were found to be 929 m^2^ g^−1^ (the Langmuir surface area is 1132 m^2^ g^−1^) and 0.612 cm^3^ g^−1^, respectively (Figure S7B, Supporting Information). Compared with nonporous TOC, the good porous structure of pTOC might have resulted from the polymeric framework that overcome the window‐to‐arene stacking of TOC and fixed the pores position to keep the permanent porosity. The BET surface area of pTOC was higher than most of porous cages,^6^, ^7^, ^19^ and comparable to some emissive porous organic polymers (POPs)^20^ (Table S1, Supporting Information). The pTOC showed a steep nitrogen uptake at low relative pressure, implying the presence of micropores in their networks. Low‐pressure hysteresis was extending to the lowest attainable pressures, which was associated with the irreversible uptake of gas molecules in the pores (or through pore entrances). This phenomenon probably means a swelling of polymer matrix at 77 K by nitrogen (**Figure** **3**A). The pore size distribution calculated using Nonlocal Density Functional Theory (NLDFT) method (Figure 3B) also confirmed the existence of micropores and mesopores, which might result from the intrinsic porosity and expanded networks of the cage monomers. It was worth noting the presence of micropores with an average size of 5.8 Å, which might have originated from the intrinsic cavity of TOC and was in agreement with the crystallographic analysis (Figure S4, Supporting Information).

**Figure 3:**
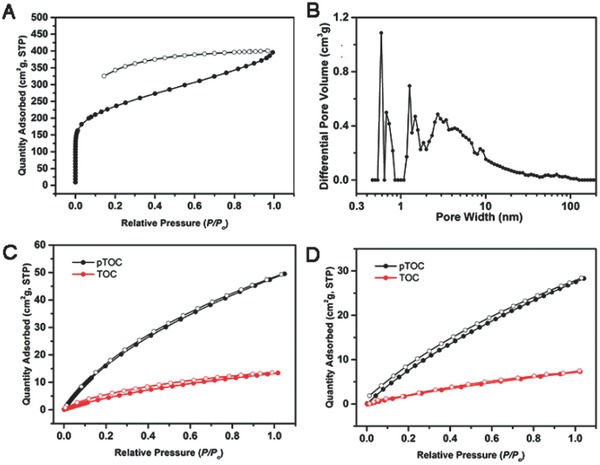
The N_2_ sorption isotherms of pTOC at 77 K A), pore size distributions of pTOC calculated using the NLDFT method B), CO_2_ sorption isotherms of TOC and pTOC at 273 K C) and 298 K D). In (A), (C), and (D), filled symbols denote gas adsorption and empty symbols denote desorption.

The CO_2_ capture experiments were performed at 273 and 298 K. As shown in Figure 3C,D, TOC can uptake CO_2_ 13.5 cm^3^ g^−1^ at 273 K/1.0 bar and 6.9 cm^3^ g^−1^ at 298 K/1.0 bar, while the pTOC showed much better CO_2_ uptake capacity of 49.3 cm^3^ g^−1^ at 273 K/1.0 bar and 28.4 cm^3^ g^−1^ at 298 K/1.0 bar. Moreover, multiple runs for CO_2_ adsorption were carried out at 273 K. The CO_2_ uptake capability of pTOC almost remained unchanged after three cycles of sorption and desorption, which demonstrated pTOC with good stability for CO_2_ adsorption (Figure S8, Supporting Information). Calculated from the adsorption isotherms at 273 and 298 K using the Clausius–Clapeyron equation^21^ (Figure S9, Supporting Information), the isosteric enthalpies (*Q*_st_) of pTOC for CO_2_ was found to be 28.3 KJ mol^−1^ that is higher than that of TOC (27.3 KJ mol^−1^). Calculated from analysis of initial of slopes of the adsorption isotherms by one single gas adsorption experiments at 273 K, the selectivity of CO_2_/N_2_ for pTOC was found to be 27 that is much higher than TOC with 9 (Figure S10, Supporting Information).

Using cost‐effective fluorescent technology of emissive porous polymers might provide a new strategy to sense CO_2_. With TPE units in scaffold, pTOC is emissive under excitation of UV light at 365 nm (Figure S11, Supporting Information). The CO_2_ sensing experiments were conducted by bubbling CO_2_ into the methanol dispersion systems of pTOC (0.3 mg mL^−1^) at room temperature and followed by fluorescence measurements. By treating with CO_2_, the fluorescent intensity of pTOC increased to 211% after 1 min and reached to 307% after 5 min (**Figure** **4**A; Figure S12, Supporting Information). For pTOC, there were abundant micropores with an average size of 5.8 Å from the well‐defined cavities of cage units, making CO_2_ molecules with the size of 3.3 Å accessible. In other words, CO_2_ can penetrate into the cavities in the pTOC scaffold and interact with nitrogen or oxygen atoms in the cage skeleton through local‐dipole/quadrupole interactions, and furthermore hinder the rotation and vibration of phenyl rings in the TPEs, which causes the radiative decay process to become predominant and increase the fluorescent intensity.^22^ Moreover, we could not observe the fluorescent changes when bubbling CO_2_ into methanol system of TOC. Their solid‐state assembly of window‐to‐arene stacking might cause the intrinsic pores blockage that leads to the CO_2_ molecules that can only be absorbed in the external channels but not in the intrinsic pores of TOC, which could not hinder the rotation and vibration of phenyl rings in the TPEs to change their fluorescent properties (Figure S13, Supporting Information). As shown in Figure 4B and Figure S12 in the Supporting Information, no change of fluorescent intensity of pTOC in methanol systems was observed after bubbling N_2_ for 5 min, which might result from the too large size of N_2_ (3.64 Å) for the pores or the weak interaction between N_2_ and pTOC. Taking the practical application into consideration, it is important to quantify the fraction of CO_2_ (*f*_CO2_) in a gas mixture. CO_2_/N_2_ mixtures were used to examine the change in fluorescence of pTOC with variations in *f*_CO2_. The CO_2_/N_2_ mixtures (10 mL) with different CO_2_ contents were bubbled into pTOC in methanol at a fixed rate. As shown in Figures S14 and S15 in the Supporting Information, the fluorescent intensity of pTOC was increased with increasing amount of CO_2_ in a linear fashion over the whole tested concentration range, which enables quantitation of CO_2_ under various conditions.

**Figure 4:**
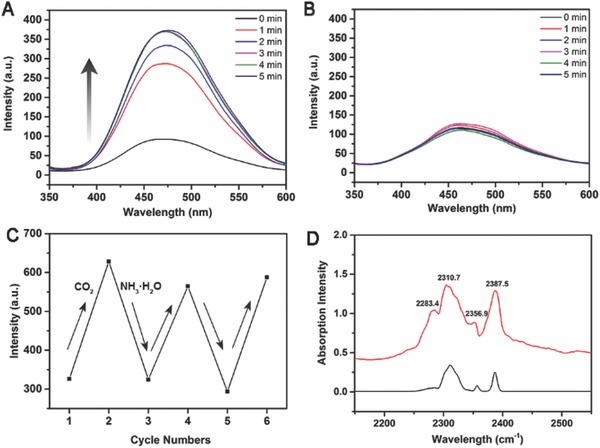
The fluorescence spectra of pTOC by bubbling CO_2_ A) and N_2_ B) during 0–5 min, the recycling tests of pTOC in MeOH upon bubbling CO_2_ and addition of NH_3_·H_2_O C) (Exited wavelength λ = 310 nm), and the FT‐IR spectra of CO_2_ sealed in a KBr cell before (black) and after (red) pTOC was loaded for 5 min.

No fluorescence enhancement occurred when hydrochloric acid was added into the methanol system of pTOC, which excluded the pH effect. To further confirm that the CO_2_ plays a key role in fluorescence enhancement of pTOC, aqueous solution of NH_3_·H_2_O was added to eliminate CO_2_. As shown in Figure 4C, the fluorescence intensity was reduced to the initial value after the addition of NH_3_·H_2_O. Furthermore, an excessive NH_3_·H_2_O did not cause any change in the fluorescence intensity of pTOC (Figure S16, Supporting Information). Recycling tests displayed that pTOC could be used repeatedly to sense CO_2_ and no significant decrease in CO_2_ detection sensitivity was observed. Moreover, heating the methanol dispersion systems of pTOC can also remove CO_2_ completely (Figure S17, Supporting Information). The phenomena of fluorescence enhancement in the presence of CO_2_ and fluorescence recovery for CO_2_ release for pTOC can also be observed in other dispersion systems such as water, ethanol, and isopropanol (Figure S18, Supporting Information). To further explore the interactions between CO_2_ molecules with the scaffold of pTOC, FT‐IR studies were also conducted. As shown in Figure 4D, after pTOC was exposed to CO_2_, a new peak was appeared obviously at 2283.4 cm^−1^ which was different from free CO_2_ peaks (2310.7, 2358.9, and 2387.5 cm^−1^) that may be ascribed to the electron‐rich nitrogen atoms facilitating the dipole‐quadrupole interactions of CO_2_ in the rigid intrinsic cavities of pTOC. These results suggested that CO_2_ molecules entrapped in the intrinsic cavity hindered rotation and vibration of phenyl rings in TPE scaffold, and thus enhanced the fluorescence of pTOC, which further validate AIE effect mechanism of restriction of intramolecular rotations.^13^

In summary, we have designed and synthesized a cage‐based emissive polymeric framework pTOC using TPE‐based oxacalixarene molecular cage TOC as monomer. It was found that pTOC performed enhanced porous properties including enhanced BET surface area, pore volume, and CO_2_ capture compared with TOC. By virtue of its strong fluorescence and the shape‐persistent intrinsic cavity, pTOC displayed prominent fluorescence enhancement in response to CO_2_ in different dispersion systems and fluorescence recovery for CO_2_ release in the presence of NH_3_·H_2_O, and it could be used repeatedly. Our synthetic protocol for emissive porous polymer with intrinsic cavity from molecular cages not only overcame the window‐to‐arene packing modes of cages to turn on their pores, but also maintained cages' intrinsic fluorescent properties, which are beneficial for the simultaneous sensing and gas storage applications.

## Conflict of Interest

The authors declare no conflict of interest.

## Supporting information

Supplementary
