# Supplementary material for: Networked Cages for Enhanced CO2 Capture and Sensing
Source: Adv Sci (Weinh). 2018 May 17;5(7):1800141. doi: 10.1002/advs.201800141 (PMC6051374; doi:10.1002/advs.201800141)
Supplement: Supplementary file 1 — Supplementary [file ADVS-5-1800141-s001.pdf]

Copyright WILEY-VCH Verlag GmbH & Co. KGaA, 69469 Weinheim, Germany, 2018.

ADVANCED SCIENCE Open Access

Supporting Information

for *Adv. Sci.,* DOI: 10.1002/advs.201800141

Networked Cages for Enhanced CO_2 Capture and Sensing

Zhen Wang, Hui Ma, Tian-Long Zhai, Guang Cheng, Qian Xu, Jun-Min Liu, Jiakuan Yang, Qing-Mei Zhang, Qing-Pu Zhang, Yan-Song Zheng,* Bien Tan,* and Chun Zhang**

Copyright WILEY-VCH Verlag GmbH & Co. KGaA, 69469 Weinheim, Germany, 2016.

Supporting Information

Networked Cages for Enhanced CO_2 Capture and Sensing

Zhen Wang, Hui Ma, Tian-Long Zhai, Guang Cheng, Qian Xu, Jun-Min Liu,, Jiakuan Yang, Qing-Mei Zhang, Qing-Pu Zhang, Yan-Song Zheng, * Bien Tan,* and Chun Zhang**

1. General information.

Materials obtained commercially were used without further purification. ^{1}H NMR and ^{13}C NMR spectra were recorded on a DMX600 NMR. MALDI-TOF mass spectra were obtained on a BIFLEXIII mass spectrometer. UV spectra were recorded on SHIMADZU UV-2041PC spectrometer. Emission spectra were obtained on HITACHI F-4500 spectrometer. The X-ray intensity data were collected on a standard Bruker SMART-1000 CCD Area Detector System equipped with a normal-focus molybdenum-target X-ray tube (λ = 0.71073 Å) operated at 2.0 kW (50 kV, 40 mA) and a graphite monochromator. The structures were solved by using direct methods and were refined by employing full-matrix least-squares cycles on F^2 (Bruker, SHELXTL-97). Surface areas and pore size distributions were measured by nitrogen adsorption and desorption at 77 K using a Micromeritics ASAP 2020 volumetric adsorption analyzer. Sample was degassed at 120 °C for 10 h under vacuum

before analysis. H_2 isotherms were measured at 77 K up to 1.0 bar using a Micromeritics ASAP 2020 volumetric adsorption analyzer with the same degassing procedure. CO_2 isotherms were measured at 273 and 298 K up to 1.0 bar using a Micromeritics ASAP 2020 volumetric adsorption analyzer with the same degassing procedure.

Atomistic Simulations. Molecular models were generated from X-ray crystallographic data structure using Materials Studio 7.0 (Accelrys). Connolly surfaces were calculated by rolling a probe molecule across the substrate, the interface taken from the contact point of the probe molecule.

2. Experimental details

Synthesis of TPE based tricyclooxacalixarene cage TOC.

Scheme S1. Synthesis of TPE-based tricyclooxacalixarene cage **TOC**.

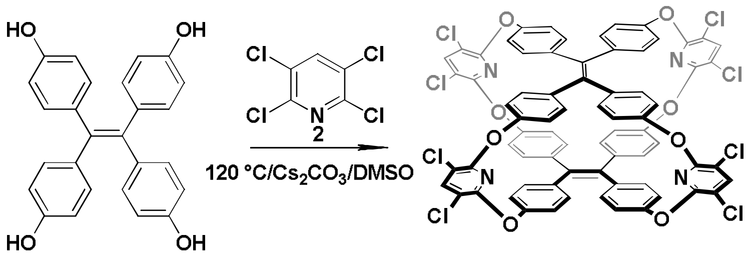

Tetra(4-hydroxyphenyl)ethylene **1** (1.00 g 2.52 mmol), 2,3,5,6-Tetrachloropyridine **2** (1.09 g, 5.05 mmol) and Cs_2CO_3 (4.92 g, 15.2 mmol) were added to a 100 mL round bottom flask. DMSO (25 mL) was added, and then the combined mixture was stirred vigorously at 120 °C overnight. After the raw materials was consumed, the reaction was allowed to cool down to RT, and the mixture was partitioned between CH_2Cl_2 (40 mL) and H_2O (40 mL), separated, the aqueous layer was extracted twice with CH_2Cl_2 (20 mL). The combined organics were dried over anhydrous Na_2SO_4, filtered, and concentrated in vacuum. The residue was purified by column chromatography (petroleum

ether/CH_2Cl_2, 5/1) to afford **TOC** as white solid (250 mg, 15 %). MALDI-TOF-MS: m/z 1368.2 (M^+). Anal. Calcd for $C_{72}H_{36}Cl_8N_4O_8$: C, 63.18; H, 2.65; N, 4.09; Found: C, 63.43; H, 2.98; N, 4.33.

Crystallographic data for **TOC**: *Mr* = 1368.65, Orthorhombic, Space group *C 12/c1*, a = 30.071(7), b = 18.602(4) Å, c = 28.613(6) Å, α = 90°, β = 16.067(3)°, γ = 90°, V = 14377(5) Å^3, Z = 8, $\rho_{calcd.}$ = 1.265 Mg/m^3, μ = 0.368 mm^{-1}, reflections collected 40861, data/restraints/parameters 12920/197/884, GOF on F^2 1.965, final R_1 = 0.1388, wR_2 = 0.3512, R indices (all data): R_1 = 0.1596, wR_2 = 0.3615, largest diff. peak and hole: 0.795 and -0.596 e/Å^{-3}, CCDC- 1525731.

Scheme S2. Synthesis of **pTOC**.

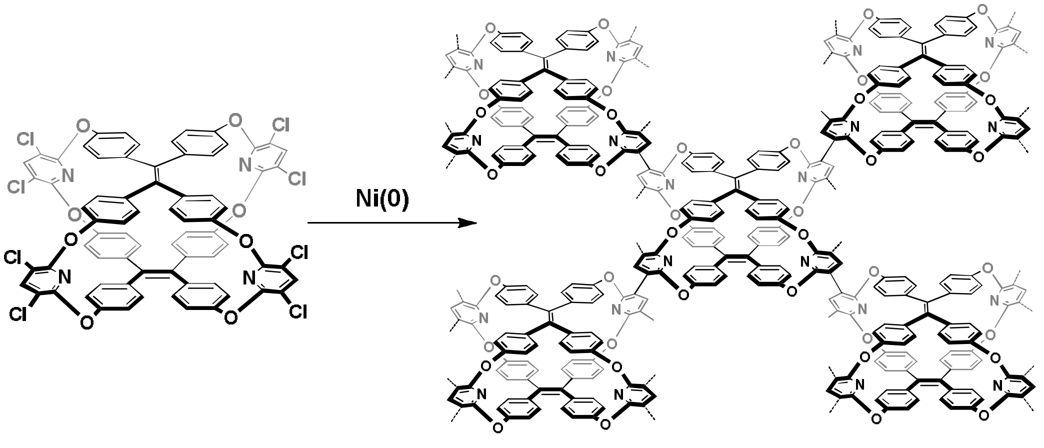

Under a dry argon atmosphere, **TOC** (200 mg, 0.15 mmol), 2-2'-bipyridyl (275 mg. 1.75 mmol) and Bis(1,5-cyclooctadiene) nickel (481 mg, 1.75 mmol) were added to a 100 mL two-neck round bottom flask, followed by a solution of 1,5 - cyclooctadiene (189 mg, 1.75 mmol) in 30 mL DMF added by syringe. The combined mixture was stirred at 85 °C for 5 d before the reaction was allowed to cool down to RT, 40 mL 2M HCl was added into the reaction solution, stirred for 0.5 h, the precipitated polymer was collected by filtration and washed with a large amount of water, the products were successively washed with excess THF and CH_2Cl_2, and then sequentially purified by Soxhlet

extraction with methanol, the purified products were dried in a vacuum oven at 60 °C for 12 h to obtain the final **pTOC** (140 mg, 88 %).

3. Variable-temperature ^{1}H NMR spectra of TOC.

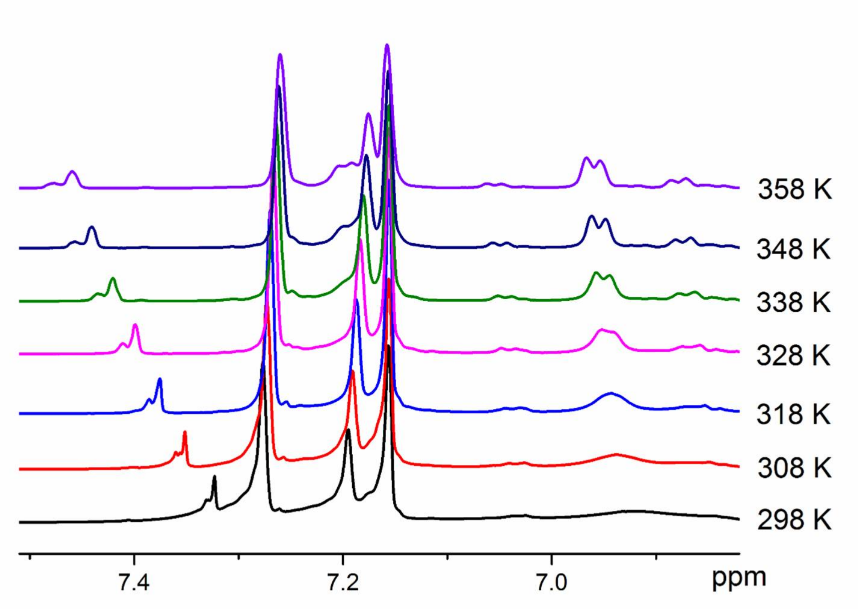

Figure S1. Variable-temperature ^{1}H NMR spectra of **TOC** in d_8-Toluene.

4. UV absorption spectra of TOC.

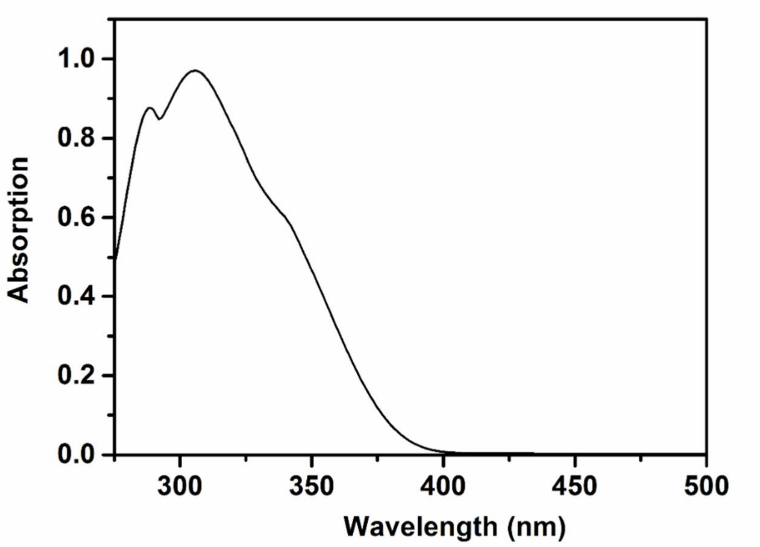

Figure S2. The UV absorption spectra of **TOC** in toluene (c = 5.0 × 10^{-5} M, 298 K).

5. Fluorescence spectra of TOC.

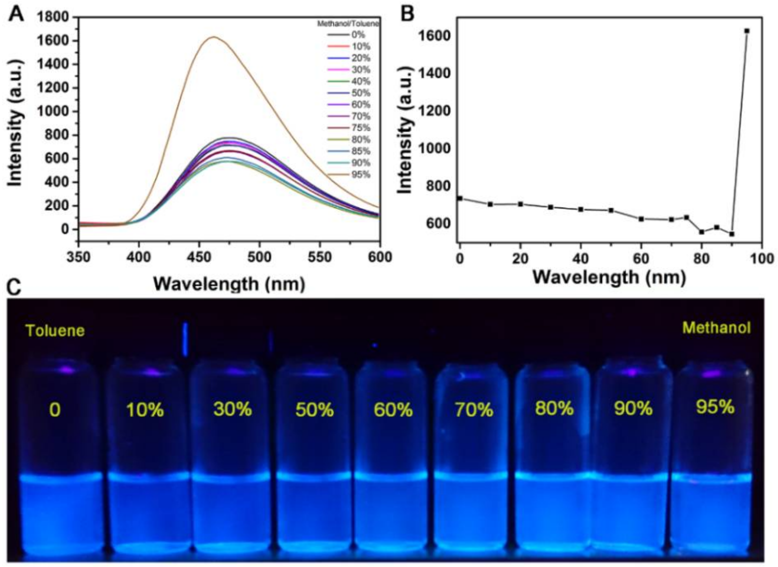

Figure S3. The fluorescence spectra of **TOC** in toluene after the addition of various amounts of methanol at the concentration of 5.0 × 10^{-6} M (excitation wavelength: 310 nm) (A). Variations of fluorescent intensity at 470 nm (B) and photos under UV irradiation (C) of **TOC** with methanol fractions (V/V) in toluene/methanol mixture.

6. X-ray crystal structures of TOC.

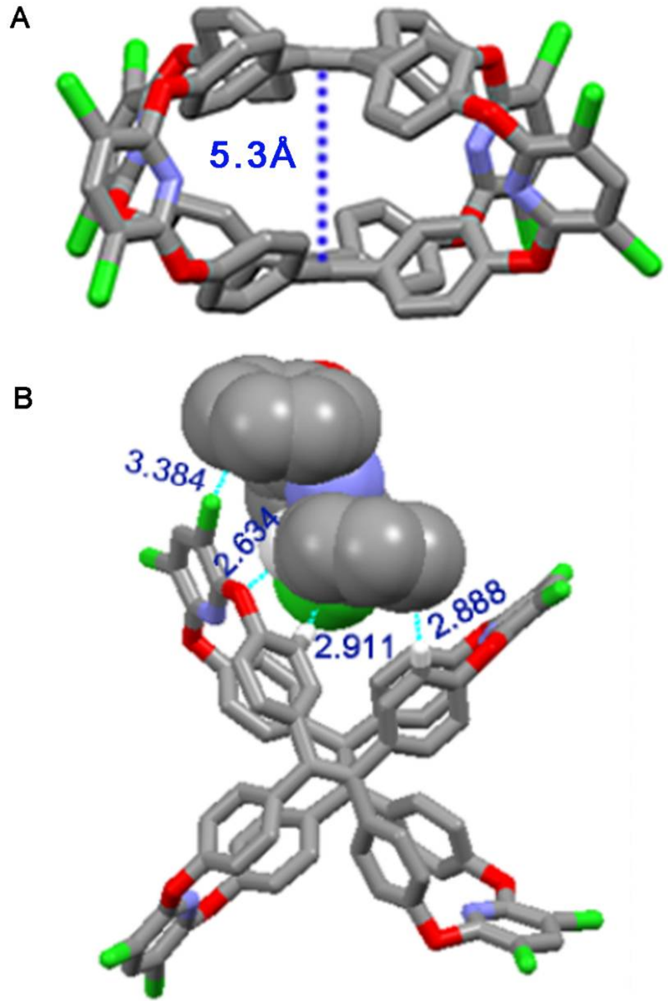

Figure S4. The X-ray crystal structure of **TOC** from the side view (A) and two neighboring molecules of **TOC** pack in a window-to-arene mode (B) (part of no contacts atoms of one neighboring molecule of **TOC** were omitted for clarity).

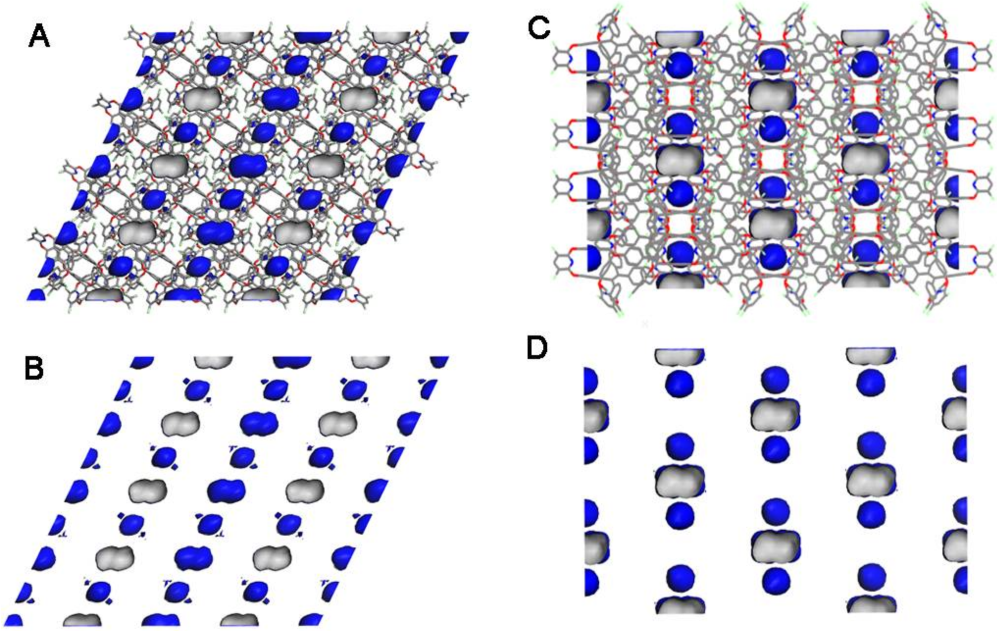

Figure S5. The Connolly surface (probe radius = 1.82 Å) of **TOC** applied to the crystal structure for the desolvated material. The images of the packing structures of **TOC** with and without cage framework from the top view (A and B) and side view (C and D).

7. FT-IR spectra of TOC and pTOC.

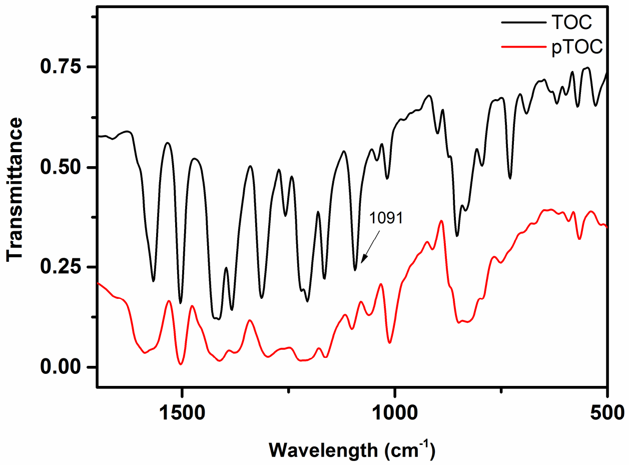

Figure S6. The FT-IR spectra of **TOC** and **pTOC**.

8. Brunauer-Emmett-Teller (BET) surface areas of TOC and pTOC.

The calculation of specific surface areas (S_{BET}) were based on the BET equation in its linearised from,

$$\frac{\frac{P}{P_0}}{V_{ads}\left(1-\frac{P}{P_0}\right)} = \frac{1}{V_m \cdot C_{BET}} + \frac{C_{BET}-1}{V_m \cdot C_{BET}} \times \frac{P}{P_0} \quad \text{(Equation S1)}$$

with P/P_0 the relative pressure; V_{ads} the adsorbed volume; V_m the monolayer volume and the constant C_{BET}.

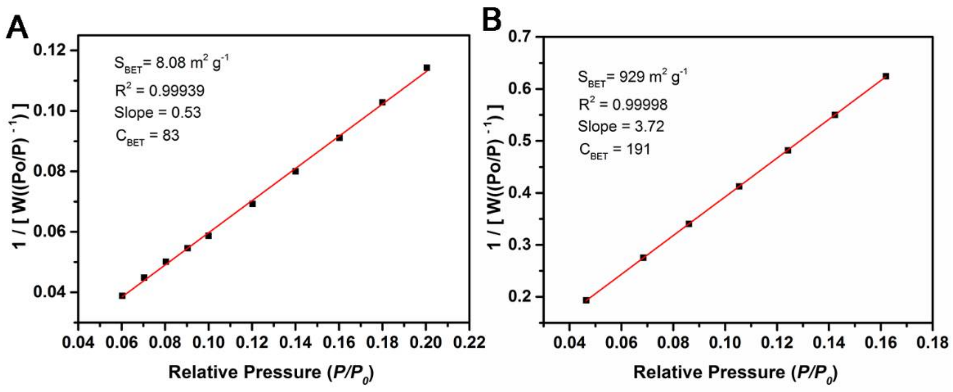

Figure S7. The Brunauer-Emmett-Teller (BET) surface areas of **TOC**(A) and **pTOC**(B) calculated from nitrogen sorption analysis at 77K.

9. Multiple runs of pTOC for CO_2 adsorption at 273K.

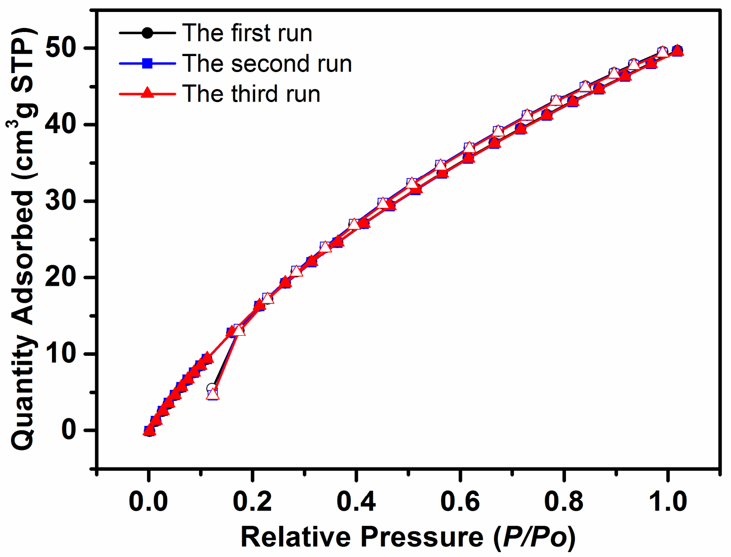

Figure S8. Three runs of **pTOC** for CO_2 adsorption at 273K.

10. Comparison of the BET surface areas of pTOC with other porous cages or porous organic polymers (POPs).

Table S1. Comparison of the BET surface areas of **pTOC** with other porous cages or porous organic polymers (POPs).

Sample	BET surface area ($m^2\ g^{-1}$)	Author
**TOC**	8	Our work
**pTOC**	929	Our work
**Cage 1-4**	Less than 10.	[1]
**Framework F1-F3**	Less than 10.	[1]
**NC1**	802	[2]
**Na-NC1**	1230	[2]
**Cage 1**	1181	[3a]
**3D-Py-COF**	1290	[3b]
**COF-BPDA**	448	[4]
**COF-TPDA**	84	[4]
**SIOC-COF-1**	478	[4]
**SIOC-COF-2**	46	[4]

[1] T. Tozawa, J. T. A. Jones, S. I. Swamy, S. Jiang, D. J. Adams, S. Shakespeare, R. Clowes, D. Bradshaw, T. Hasell, S. Y. Chong, C. Tang, S. Thompson, J. Parker, A. Trewin, J. Bacsa, A. M. Z. Slawin, A. Steiner, A. I. Cooper, *Nat. Mater*. **2009**, *8*, 973.

[2] Y. H. Jin, B. A. Voss, R. McCaffrey, C. T. Baggett, R. D. Nobleb, W. Zhang, *Chem. Sci.* **2012**, *3*, 874.

[3] L. Zhang, L. Xiang, C. Hang, W. L. Liu, W. Huang, Y. C. Pan, *Angew. Chem. Int. Ed.* **2017**, *56*, 7787.

[4] a) H. M. Ding, Y. H. Yang, B. J. Li, F. Pan, G. Z. Zhu, M. Zeller, D. Q. Yuan, C. Wang, *Chem. Commun*. **2015**, *51*, 1976. b) G. Lin, H. Ding, D. Yuan, B. Wang, C. Wang, *J. Am. Chem. Soc.* **2016**, *138*, 3302.

[5] O. Buyukcakir, Y. Seo, A. Coskun, *Chem. Mater.*, **2015**, *27*, 4149.

[6] a) Z. F. Pang, S. Q. Xu, T. Y. Zhou, R. R. Liang, T. G. Zhan, X. Zhao, *J. Am. Chem. Soc.* **2016**, *138*, 4710; b) G. Lin, H. Ding, D. Yuan, B. Wang, C. Wang, *J. Am. Chem. Soc.* **2016**, *138*, 3302.

11. Isosteric enthalpies of adsorption for CO_2 of TOC and pTOC.

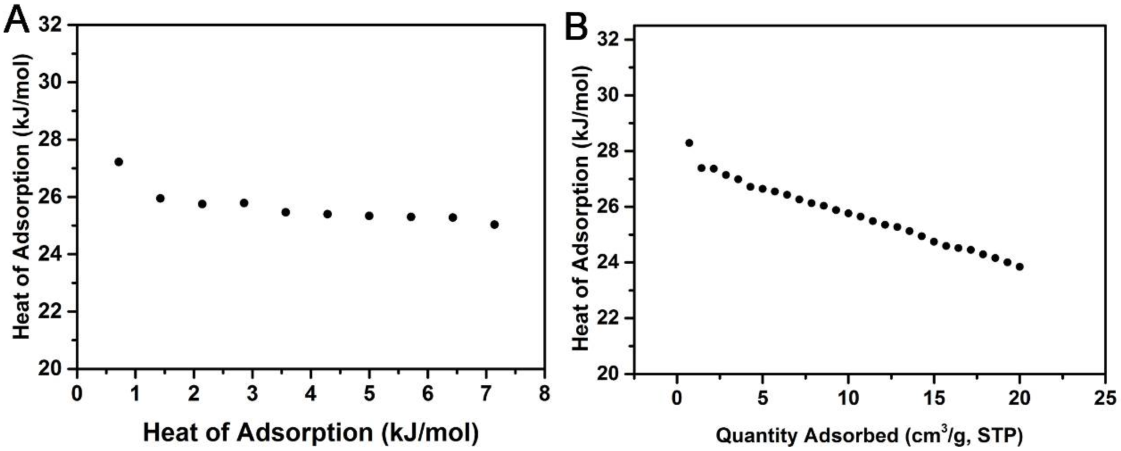

Figure S9. Isosteric enthalpies of adsorption for CO_2 of **TOC** and **pTOC**.

12. Initial gas uptake slopes of TOC and pTOC at 273K.

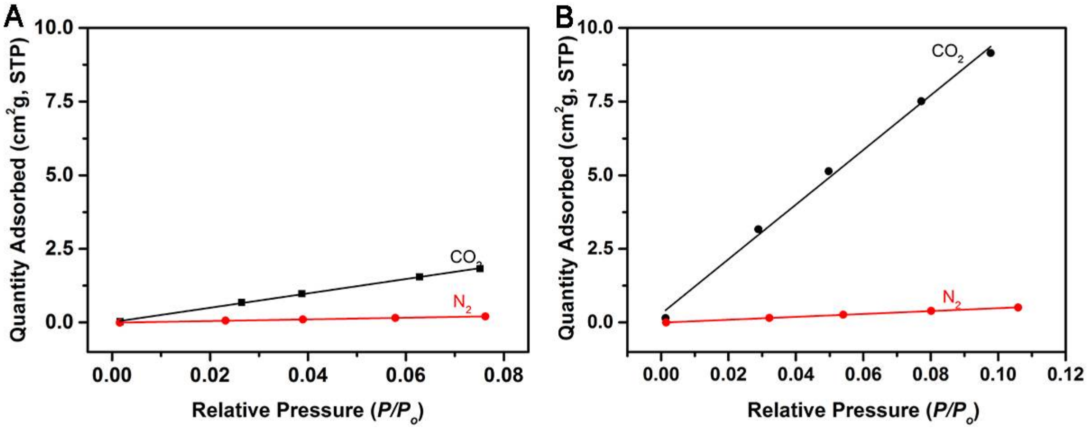

Figure S10. Initial gas uptake slopes of **TOC** and **pTOC** at 273K.

13. Photographs of pTOC dispersed in MeOH

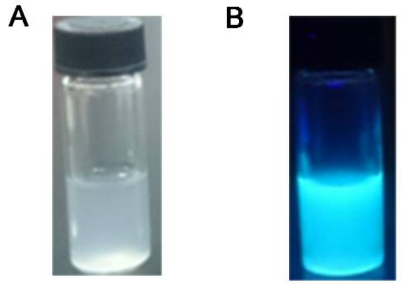

Figure S11. The images of **pTOC** dispersed in MeOH under daylight (A) and under UV lamp (365 nm) (B).

14. The fluorescent intensity variations of pTOC at 470 nm in MeOH after bubbling CO_2 and N_2.

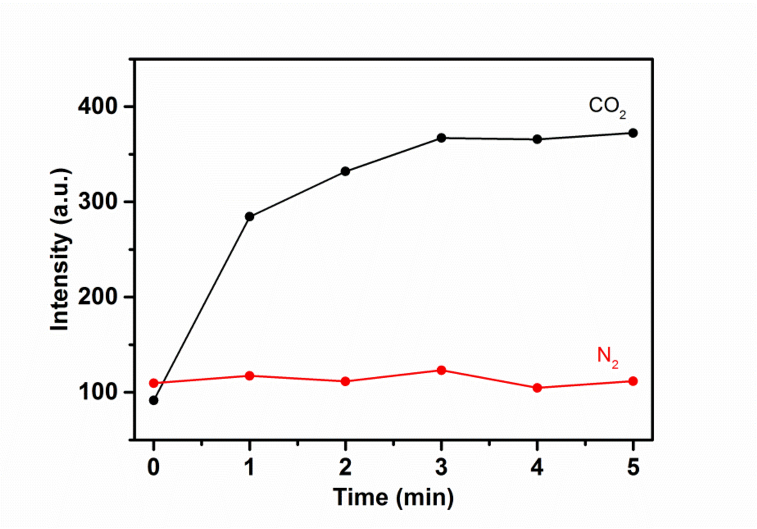

Figure S12. The fluorescent intensity variations of **pTOC** at 470 nm in MeOH after bubbling CO_2 and N_2.

15. The fluorescent intensity variations of TOC after bubbling CO_2

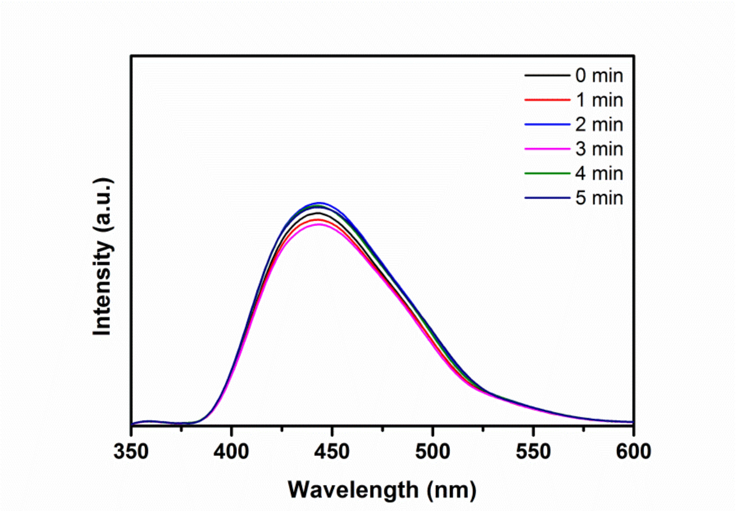

Figure S13. The fluorescent intensity variations of **TOC** in MeOH (0.3mg/mL) by bubbling CO_2 for 5min.

16. Fluorescent intensity variations of pTOC after bubbling the CO_2/N_2 mixtures with different CO_2 contents.

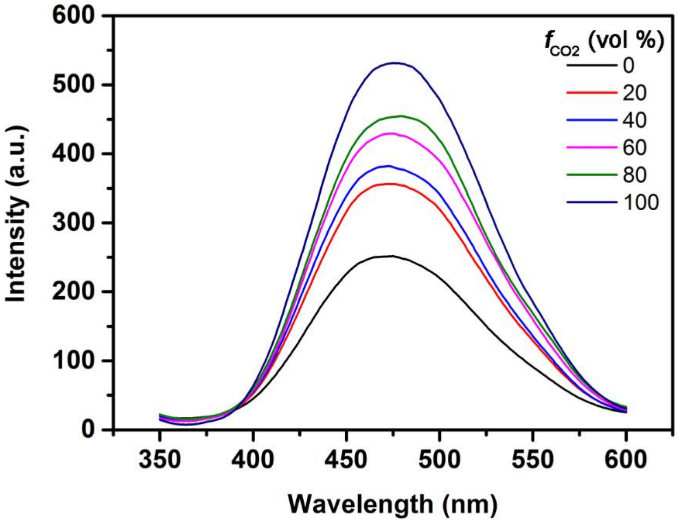

Figure S14. The fluorescent spectra of **pTOC** in MeOH after bubbling different fractions CO_2 (f_{CO2}) in CO_2/N_2 mixtures.

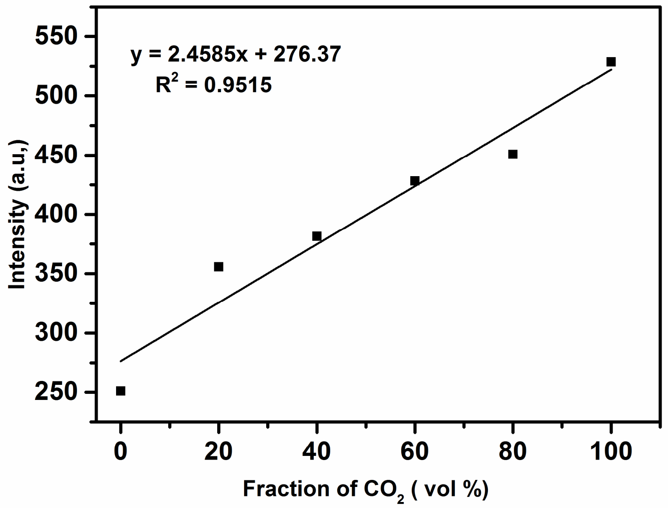

Figure S15. Plots of fluorescent intensities (470nm) of **pTOC** in MeOH versus fractions CO_2 (f_{CO2}) in CO_2/N_2 mixtures.

17. Fluorescence intensity of pTOC in MeOH after adding $NH_3 \cdot H_2O$.

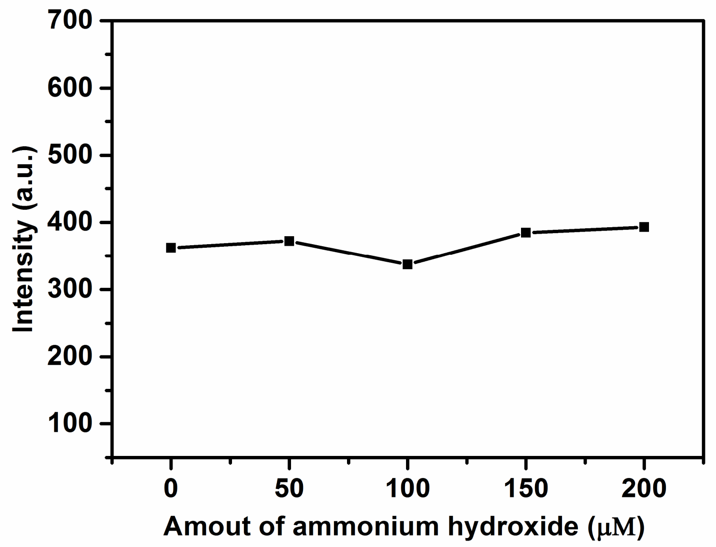

Figure S16. The fluorescent intensity variation of **pTOC** in MeOH by addiation of $NH_3 \cdot H_2O$.

18. Cycling tests of pTOC in MeOH upon bubbling with CO_2 and heating.

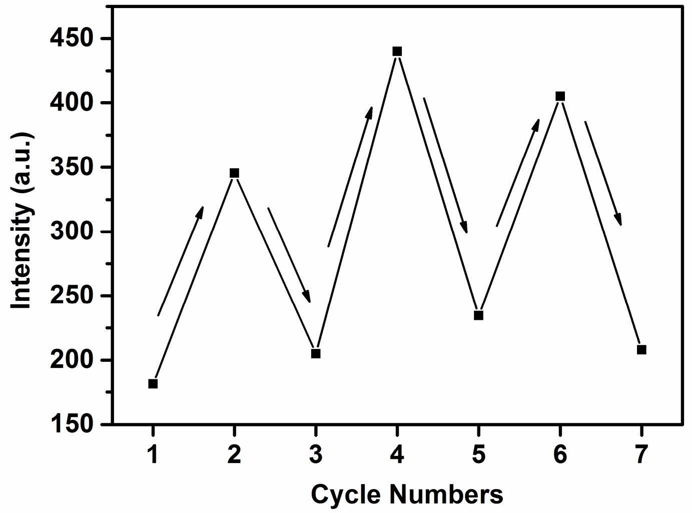

Figure S17. The cycling tests of **pTOC** in MeOH upon bubbling with CO_2 and heating.

19. The fluorescence spectrum of pTOC after bubbling CO_2 and heating in water.

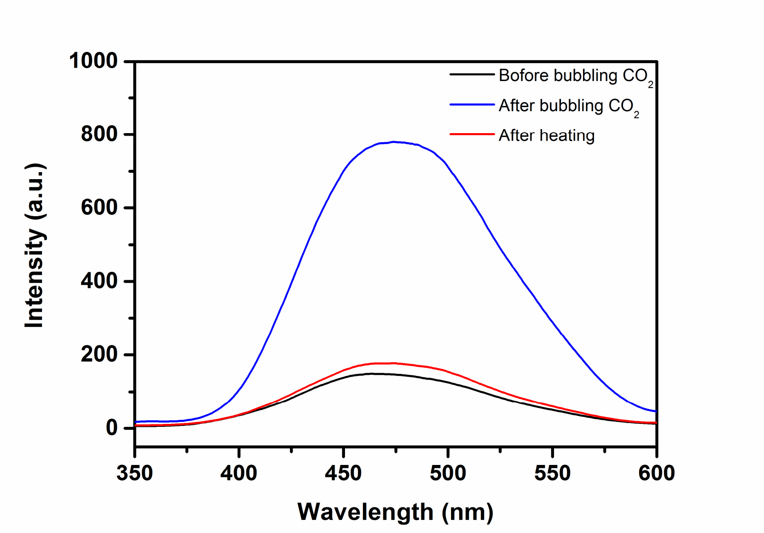

Figure S18. The fluorescence spectra of **pTOC** after bubbling CO_2 and heating in water.
